# Twelve-Week Protocatechuic Acid Administration Improves Insulin-Induced and Insulin-Like Growth Factor-1-Induced Vasorelaxation and Antioxidant Activities in Aging Spontaneously Hypertensive Rats

**DOI:** 10.3390/nu11030699

**Published:** 2019-03-25

**Authors:** Kunanya Masodsai, Yi-Yuan Lin, Rungchai Chaunchaiyakul, Chia-Ting Su, Shin-Da Lee, Ai-Lun Yang

**Affiliations:** 1 Institute of Sports Sciences, University of Taipei, Taipei 11153, Taiwan; kunanyamasodsai@gmail.com; 2 Department of Occupational Therapy, Asia University, Taichung 41354, Taiwan; charlet8116@gmail.com (Y.-Y.L.); leeshinda@gmail.com (S.-D.L.); 3 Graduate Institute of Clinical Medical Science, China Medical University, Taichung 40402, Taiwan; 4 College of Sports Science and Technology, Mahidol University, Nakhonpathom 73170, Thailand; gmrungchai@gmail.com; 5 Department of Occupational Therapy, College of Medicine, Fu Jen Catholic University, New Taipei City 24205, Taiwan; chiatingsu@gmail.com; 6 Department of Physical Therapy, Graduate Institute of Rehabilitation Science, China Medical University, Taichung 40402, Taiwan; 7 School of Rehabilitation Science, Shanghai University of Traditional Chinese Medicine, Shanghai 201203, China

**Keywords:** polyphenol, high blood pressure, elderly, endothelium, nitric oxide

## Abstract

Protocatechuic acid (PCA), a strong antioxidant, has been reported for its cardiovascular-protective effects. This study aimed to investigate the effects of PCA administration on vascular endothelial function, mediated by insulin and insulin-like growth factor-1 (IGF-1), and antioxidant activities in aging hypertension. Thirty-six-week-old male aging spontaneously hypertensive rats were randomly divided into vehicle control (SHR) and PCA (SHR+PCA) groups, while age-matched Wistar–Kyoto rats (WKY) served as the normotensive vehicle control group. The oral PCA (200 mg/kg/day) was administered daily for a total of 12 weeks. When the rats reached the age of 48 weeks, the rat aortas were isolated for the evaluation of vascular reactivity and Western blotting. Also, nitric oxide (NO) production and antioxidant activities were examined among the three groups. The results showed that, when compared with the SHR group, the insulin-induced and IGF-1-induced vasorelaxation were significantly improved in the SHR+PCA group. There was no significant difference in the endothelium-denuded vessels among the three groups. After the pre-incubation of phosphatidylinositol 3-kinase (PI3K) or NO synthase (NOS) inhibitors, the vasorelaxation was abolished and comparable among the three groups. The protein levels of insulin receptors, IGF-1 receptors, phospho-protein kinase B (p-Akt)/Akt, and phospho-endothelial NOS (p-eNOS)/eNOS in aortic tissues were significantly enhanced in the SHR+PCA group when compared with the SHR group. Moreover, significant improvements of nitrate/nitrite concentration and antioxidant activities, including superoxide dismutase, catalase, and total antioxidants, were also found in the SHR+PCA group. In conclusion, the 12 weeks of PCA administration remarkably improved the endothelium-dependent vasorelaxation induced by insulin and IGF-1 in aging hypertension through enhancing the PI3K–NOS–NO pathway. Furthermore, the enhanced antioxidant activities partly contributed to the improved vasorelaxation.

## 1. Introduction

Hypertension is recognized as a risk factor for cardiovascular disease (CVD). The prevalence of hypertension has been dramatically increasing worldwide, especially in older adults and the elderly. More than 60% of the population above the age of 65 suffer from hypertension [1]. In older adults with hypertension, more profound pathological processes may exist to induce CVD. Hypertension leads to reversible cardiac and vascular dysfunction, including cardiac hypertrophy, reduced lumen diameter of arteries, increased vascular smooth muscle cell (VSMC) proliferation, and endothelial dysfunction [2,3,4]. Moreover, in aging heart and vessels, the number of myocardial cells is decreased, cardiac fibrosis is increased, elastic arteries become stiffer, and endothelial function is impaired [5,6]. Since vascular endothelium is critical for homeostasis in the body, its impairment evokes pathological conditions, such as hypertension, diabetes, and CVD (e.g., atherosclerosis, thrombosis) [7]. The impaired bioavailability of nitric oxide (NO) and imbalance of endothelium-derived vasoconstrictive and vasodilatory substances are considered as the key features of endothelial dysfunction. Furthermore, a reduction in activity of antioxidant enzyme, such as superoxide dismutase (SOD), has been suggested to be another mechanism for endothelial dysfunction, which is also associated with cardiac and vascular aging [8,9,10]. It has been known that insulin and insulin-like growth factor 1 (IGF-1) have important vascular actions that stimulate NO production mainly in the endothelium. They both modulate the endothelium-dependent vasorelaxation by activating phosphatidylinositol 3-kinase (PI3K), protein kinase B (PKB/Akt), and endothelial nitric oxide synthase (eNOS), which results in NO production [11,12,13]. Previous research has reported that the vascular actions of insulin and IGF-1 are pathologically altered and impaired in cardiovascular disorders, such as hypertension and obesity. Also, the diminished vasorelaxant effects of insulin and IGF-1 have been found in the development of hypertension [14,15,16,17]. However, in aging hypertension, the ways insulin and IGF-1 influence the cardiovascular dysfunction remain unclear.

Numerous strategies in prevention and/or treatment for ameliorating high blood pressure have been widely prescribed, such as lifestyle and dietary modifications (e.g., low-sodium intake, and fruit- and vegetable-enriched consumption) [18]. Protocatechuic acid (PCA, 3,4-dihydroxybenzoic acid), a strong antioxidant, is a natural phenolic compound found in many types of food. It has been known for multiple benefits to health, including anti-inflammation, anti-hyperglycemic, anti-hypertensive, and cardiovascular-protective effects [19,20,21]. A previous study indicated significant preservation of increasing blood pressure and improved antioxidant capacity in dexamethasone-induced hypertensive rats by short-term daily administration of 200 mg/kg PCA [22]. Also, a single dose of intraperitoneal injection of alpinia PCA (5 and 10 mg/kg) for 7 days in aged rats remarkably improved antioxidant activities (i.e., glutathione peroxidase and catalase) and suppressed oxidative stress (malondialdehyde, MDA) [23]. Additionally, administering various doses of PCA decreased blood glucose and hemoglobin A1c (HbA1_C_) in studies of anti-hyperglycemic effects [24,25]. However, the effectiveness of PCA administration on aging hypertension with regard to the vasorelaxant effects of insulin and IGF-1 and antioxidant activities has not been fully investigated. Therefore, in the present study, we investigated the effects of PCA administration on insulin-induced and IGF-1-induced vasorelaxation and antioxidant activities in aging spontaneously hypertensive rats (SHR).

## 2. Materials and Methods

### 2.1. Animals

Eight-week-old male SHR and Wistar–Kyoto rats (WKY) were purchased from the National Laboratory Animal Center (Taipei, Taiwan). All rats were attentively housed in an environment-controlled room at 22–24 °C with a 12-h dark/light cycle (lights on at 06:00 h and off at 18:00 h) and provided standard laboratory chow (Lab Diet 5001; PMI Nutrition International, Brentwood, MO, USA) and water ad libitum. At the age of 36 weeks, the SHR were randomly divided into the vehicle control group (SHR, *n* = 8) and the protocatechuic acid (PCA)-treated group (SHR+PCA, *n* = 8). The age-matched WKY served as the normotensive vehicle control group (WKY, *n* = 8). The SHR+PCA group was treated with 200 mg/kg body weight of PCA (Sigma Chemical, St. Louis, MO, USA), dissolved in daily water, for a total of 12 weeks [22,24]. The other groups were not treated with PCA in their daily water. The rats in the SHR+PCA group were provided with the PCA-containing water in the afternoon and checked by the assistant every hour to monitor the drinking. After the rats finished drinking the PCA-containing water (2–3 h), they were provided with regular water ad libitum. They drank all of the PCA-containing water every day. All samples were collected at least 24 h after the animals finished the administration of PCA. This study was conducted in conformity under the Guide for the Care and Use of Laboratory Animals of the National Institutes of Health. All experimental procedures were approved by the Institutional Animal Care and Use Committee (IACUC) of the University of Taipei, Taiwan (Ethical approval code: UT104005).

### 2.2. Resting Blood Pressure and Heart Rate

The heart rate and systolic blood pressure (SBP) were measured noninvasively by the tail-cuff method (BP98A, Softron, Tokyo, Japan) [14]. The hemodynamic data were collected between 9:00 h and 12:00 h, at least 24 h after drinking the PCA.

### 2.3. Vasoreactivity Experiments

At the end of the experimental period, all rats were fasted overnight and were sacrificed under anesthesia with 2% isoflurane delivered in oxygen (95% O_2_ and 5% CO_2_). Then, thoracic aortas were carefully isolated. The force displacement transducers (Models FT3E, Grass Instrument, West Warwick, RI, USA) were used to isometrically evaluate the vasorelaxant responses of isolated rings which were submerged in organ chambers containing Krebs–Ringer buffer (118 mM NaCl, 4.8 mM KCl, 2.5 mM CaCl_2_, 1.2 mM MgSO_4_, 1.2 mM KH_2_PO_4_, 24 mM NaHCO_3_, 0.03 mM Na-EDTA, and 11 mM glucose; pH 7.4) oxygenated with 95% O_2_ and 5% CO_2_ at 37 °C. After the 60-min equilibration at the optimal passive tension (i.e., 2 g), the drugs were administered. The pre-contraction of aortic rings was induced by phenylephrine (10^−7^ M, Sigma Chemical) before being exposed to various concentrations of insulin (3 × 10^−8^ to 3 × 10^−6^ M) and IGF-1 (10^−9^ to 10^−7^ M, PeproTech, NJ, USA) to induce dose-dependent vasorelaxation. The endothelial integrity was confirmed by at least 60% of acetylcholine (10^−7^ M)-induced vasorelaxation in phenylephrine-precontracted vessel rings. The parallel testing of the endothelium-denuded rings was also evaluated among the three groups. The endothelium-denuded rings were indicated by less than 10% of acetylcholine (10^−7^ M)-induced vasorelaxation precontracted with phenylephrine. Moreover, to determine the roles of PI3K and NOS in the insulin- and IGF-1-induced vasorelaxant responses, the selective inhibitors, wortmannin (3 × 10^−7^ M; an inhibitor of PI3K; Sigma Chemical) and nitro-l-arginine methyl ester (l-NAME) (10^−6^ M; a NOS inhibitor; Sigma Chemical), were pre-incubated for 15 min before the administration of phenylephrine in the endothelium-intact rings. Stock solutions of all drugs were prepared and dissolved in distilled water except for insulin (dissolved in 10^−2^ M HCl). Final dilutions of the drugs were prepared in distilled water immediately before use. None of the vehicles used in final dilutions induced any significant effects on vessel tone [14].

### 2.4. Blood Collection and Biochemical Analysis

Blood samples were allowed to clot for 30 min at room temperature and then centrifuged at 2000× *g* for 15 min at 4 °C. The serum was collected after the centrifugation and stored at −80 °C for biochemical analysis. Serum nitrate/nitrite concentration was performed using a nitrate/nitrite colorimetric assay kit (Cayman Chemical Company, Ann Arbor, MI, USA). According to the manufacturer’s instructions, total nitrate/nitrite concentration was determined by a two-step process. The first step was the conversion of nitrate to nitrite by nitrate reductase. The second step was the addition of Griess Reagents which convert nitrite to a deep purple azo product. The absorbance due to the azo chromophore was measured at 450 nm by a microplate reader (TECAN Infinite M200PRO, Grödig, Austria). The concentration was expressed in μM in serum samples. Serum MDA concentration, an index of lipid peroxidation marker, was determined using a thiobarbituric acid reactive substances (TBARS) assay kit (Cayman Chemical Company). The TBA reagents prepared according to the manufacturer’s protocol was mixed with the serum samples to generate the MDA-TBA adducts under high temperature (90–100 °C) and acidic condition. After completing the reactions, samples were measured colorimetrically at 540 nm by a microplate reader (TECAN Infinite M200PRO). The concentration was expressed in μM in serum samples. Serum SOD activity was determined using a SOD assay kit (Cayman Chemical Company). The assay utilized a tetrazolium salt for the detection of superoxide radicals generated by xanthine oxidase and hypoxanthine. One unit of SOD was defined as the amount of enzyme needed to exhibit 50% dismutation of superoxide radicals. The absorbance was read at 450 nm by a microplate reader (TECAN Infinite M200PRO) and the activity was expressed in U/mL in serum samples. Serum catalase activity was performed using a catalase assay kit (Cayman Chemical Company). The assay relied on the reaction of catalase in the sample with methanol in the presence of hydrogen peroxide (H_2_O_2_). The absorbance was read at 540 nm by a microplate reader (TECAN Infinite M200PRO) and the activity was expressed in nmol/min/mL in serum samples. Serum total antioxidant capacity was performed using an antioxidant assay kit (Cayman Chemical Company). The assay was based on the ability of antioxidants in the sample to inhibit the oxidation of the 2,2′-azino-di-(3-ethylbenzothiazoline sulphonate) (ABTS) to ABTS**˙^⁺^** by metmyoglobin. The capacity of the antioxidants in the sample was compared with that of Trolox, a water-soluble tocopherol analogue, as the standard, and was quantified as millimolar (mM) Trolox equivalents. The absorbance was measured colorimetrically at 750 nm by a microplate reader (TECAN Infinite M200PRO).

### 2.5. Insulin Resistance Determination

Fasting plasma glucose was determined by the glucose oxidase method using the glucometer (Roche Diagnostics, Indianapolis, IN, USA). According to the manufacturer’s instructions, serum insulin was measured using a commercial ELISA kit (Mercodia AB, Uppsala, Sweden). Briefly, adding enzyme conjugate solution incubated with serum sample, the bound conjugate was detected by the reaction with 3,3′-5,5′-tetramethylbenzidine (TMB). The reaction was stopped by the stop solution and the absorbance was measured at 450 nm by a microplate reader (TECAN Infinite M200PRO). The homeostatic model assessment of insulin resistance (HOMA-IR) was calculated by the equation: HOMA-IR = (fasting glucose (mmol/L) × fasting serum insulin (mU/L))/22.5 [26].

### 2.6. Western Immunoblotting

Western immunoblotting analysis was performed as previously described [14]. Aortic tissue extracts from thoracic aortas were obtained by homogenizing at 4 °C in the tissue protein extraction reagent (T-PER, Thermo Scientific) supplemented with complete protease and phosphatase inhibitors (Sigma Chemical). The supernatant was collected after sequential centrifuge of the homogenates at 10,000 rpm for 10 min. Protein concentration of the aortic tissue extract supernatant was determined by the Bradford method (Bio-Rad Laboratories, Hercules, CA, USA) with bovine serum albumin as a standard. Protein samples (50 µg/lane) were separated by 8% sodium dodecyl sulfate-polyacrylamide gel electrophoresis (SDS-PAGE) with a minigel apparatus (Bio-Rad Laboratories) and subsequently transferred to polyvinylidene difluoride (PVDF) membranes (Millipore, Bedford, MA, USA). The membranes were incubated with blocking buffer (5% nonfat dry milk in tris-buffered saline and Tween 20 (TBST) buffer) for 1 h. Primary antibodies including the anti-insulin receptor, anti-IGF-1 receptor, anti-phospho-Akt, anti-Akt (diluted at a ratio 1:1000; Cell Signaling Technology, Danvers, MA, USA), anti-phospho-eNOS, anti-eNOS (diluted at a ratio 1:1000; BD Transduction Laboratories, Lexington, KY, USA), and actin (diluted at a ratio 1:5000; Millipore) were diluted in antibody-binding buffer overnight at 4 °C. After incubation with the appropriate primary antibody, the peroxidase-conjugated secondary antibodies (1:5000; Millipore) were then incubated at room temperature for 1 h. The immunoblotted proteins were detected by enhanced chemiluminescence by using enhanced chemiluminescence detection reagents in the Gel Doc XR System (Bio-Rad Laboratories).

### 2.7. Statistical Analysis

All data were presented as means ± standard error (SE). Estimated parameters were compared among the WKY, SHR, and SHR+PCA groups using one-way ANOVA with pre-planned contrast comparison with the control group and then LSD post hoc analysis. Dose responses of vasorelaxation were analyzed by two-way ANOVA with a repeated measures design (using SPSS software v.21). For all statistical tests, *p* < 0.05 was considered to be significant.

## 3. Results

### 3.1. General Characteristics

As shown in Table 1, the heart rate and SBP were significantly (*p* < 0.05) increased in the SHR group when compared with the WKY group. After 12 weeks of PCA administration, these indicators were significantly (*p* < 0.05) reduced in the SHR+PCA group compared with the SHR group. In addition, the blood glucose and insulin concentration were significantly (*p* < 0.05) increased in the SHR group when compared with WKY; however, both of them were significantly (*p* < 0.05) decreased in the SHR+PCA group when compared with the SHR. The level of insulin resistance indicated by HOMA-IR was also significantly (*p* < 0.05) augmented in the SHR group, but after the PCA intervention, it was significantly (*p* < 0.05) reduced in the SHR+PCA group. The body weight was similar among the three groups.

### 3.2. Insulin-Induced and IGF-1-Induced Vasorelaxation in Aortas

**Figure 1:**
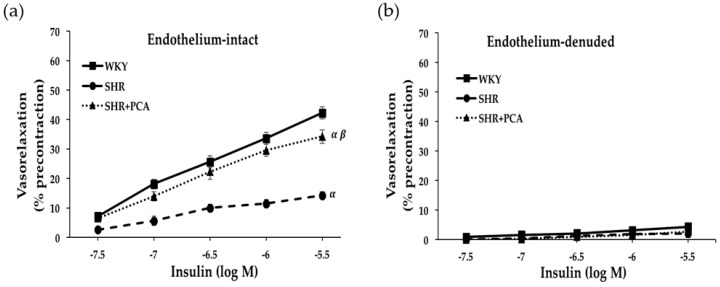
Insulin (3 × 10^−8^ to 3 × 10^−6^ M)-induced vasorelaxation at cumulative concentration–response curves in (**a**) endothelium-intact and (**b**) endothelium-denuded aortic rings among the WKY, SHR, and SHR+PCA groups. ^α^, *p* < 0.05, significant differences from WKY; ^β^, *p* < 0.05, significant differences from SHR; *n* = 8 in each group.

**Figure 2:**
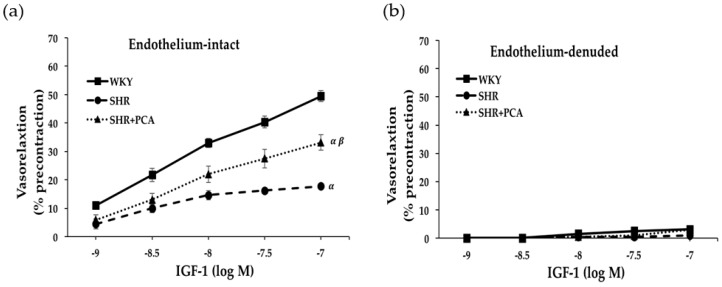
Insulin-like growth factor-1 (IGF-1; 10^−9^ to 10^−7^ M)-induced vasorelaxation at cumulative concentration–response curves in (**a**) endothelium-intact and (**b**) endothelium-denuded aortic rings among the WKY, SHR, and SHR+PCA groups. ^α^, *p* < 0.05, significant differences from WKY; ^β^, *p* < 0.05, significant differences from SHR; *n* = 8 in each group.

Figure 1 and Figure 2 revealed the concentration-response curves of insulin-induced and IGF-1-induced vasorelaxation in the isolated aortic rings in the WKY, SHR, and SHR+PCA groups. In endothelial-intact aortic rings, the SHR group had significantly (*p* < 0.05) diminished responses in insulin-induced and IGF-1-induced vasorelaxation when compared with the WKY group. However, these impaired responses were significantly (*p* < 0.05) improved in the SHR+PCA group when compared with the SHR group. As noted, the improvements did not reach the level of vasorelaxation in the WKY group. To determine the endothelium-dependent responses, the parallel investigation of endothelium-denuded of aortic rings showed that no significant difference was found in insulin-induced and IGF-1-induced vasorelaxation among the three groups.

### 3.3. Roles of PI3K and NOS in Insulin-Induced and IGF-1-Induced Vasorelaxation

**Figure 3:**
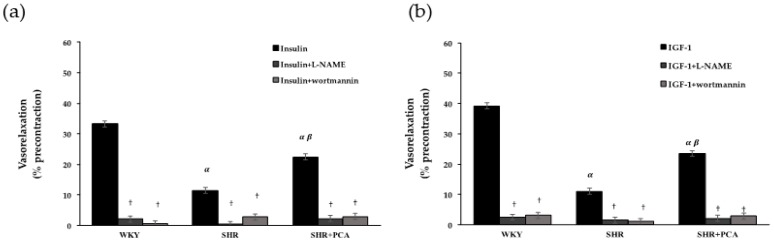
(**a**) Insulin (10^−6^ M)-induced vasorelaxation and (**b**) IGF-1 (3 × 10^−8^ M)-induced vasorelaxation after the 15-min pre-incubation with wortmannin (3 × 10^−7^ M) or nitro-l-arginine methyl ester (l-NAME) (10^−6^ M) among the WKY, SHR, and SHR+PCA groups. ^α^, *p* < 0.05, significant differences from WKY; ^β^, *p* < 0.05, significant differences from SHR; †, *p* < 0.05, significant differences from no inhibitors; *n* = 8 in each group.

The selective inhibitors, wortmannin and l-NAME, were pretreated in endothelium-intact aortic rings to verify the roles of PI3K and NOS in insulin-induced and IGF-1-induced vasorelaxation (Figure 3). Before adding wortmannin or l-NAME, the SHR group had significantly (*p* < 0.05) lower insulin-induced and IGF-1-induced vasorelaxation than the WKY group. Nevertheless, the SHR+PCA group had significantly (*p* < 0.05) higher vasorelaxant responses than the SHR group. After the 15-min pre-incubation of wortmanin or l-NAME, the insulin-induced and IGF-1-induced vasorelaxation was significantly (*p* < 0.05) blunted in all groups. Moreover, there was no significant difference in these vasorelaxant responses among the three groups.

### 3.4. Aortic Protein Expression

**Figure 4:**
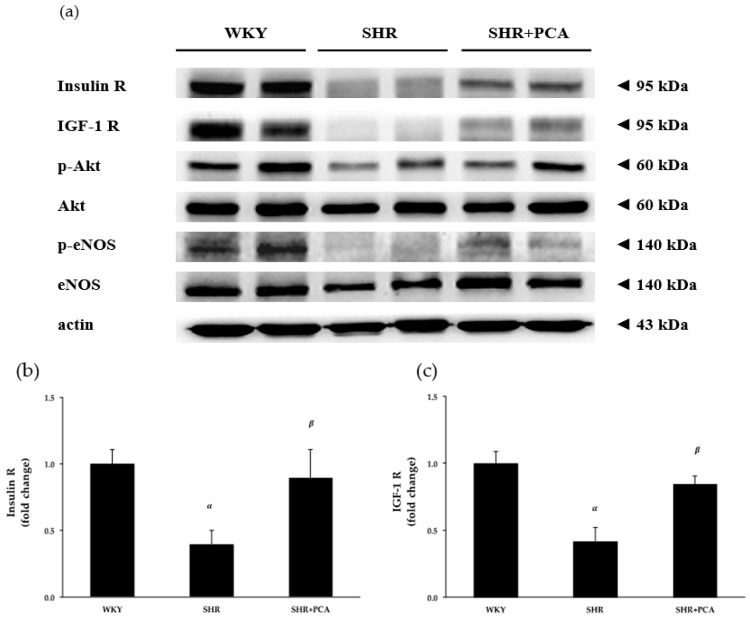

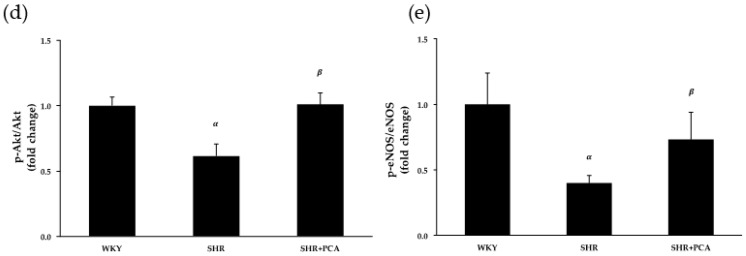
(**a**) Representative immunoblots of insulin receptor (insulin R), insulin-like growth factor-1 receptor (IGF-1 R), phospho-protein kinase B (p-Akt), protein kinase B (Akt), phospho-endothelial nitric oxide synthase (p-eNOS), endothelial nitric oxide synthase (eNOS), and actin extracted from thoracic aortas, and relative protein quantification of (**b**) insulin R, (**c**) IGF-1 R, (**d**) p-Akt/Akt, and (**e**) p-eNOS/eNOS on the basis of actin among the WKY, SHR, and SHR+PCA groups. ^α^, *p* < 0.05, significant differences from WKY; ^β^, *p* < 0.05, significant differences from SHR; *n* = 6 in each group.

To determine the effects of PCA administration on aortic protein expression involved in the insulin- and IGF-1-mediated vasorelaxation, the protein levels of insulin receptors, IGF-1 receptors, phospho-Akt (p-Akt), Akt, phospho-eNOS (p-eNOS), and eNOS extracted from thoracic aortas were evaluated by Western blotting (Figure 4 and Supplementary Figure S1). Figure 4 showed that the protein levels of insulin receptors, IGF-1 receptors, p-Akt/Akt, and p-eNOS/eNOS were significantly (*p* < 0.05) diminished in the SHR group (*p* < 0.05) when compared with the WKY group. Netherless, the PCA administration significantly (*p* < 0.05) improved these protein levels in the SHR+PCA group when compared with the SHR group.

### 3.5. Serum Nitrate/Nitrite Concentration

**Figure 5:**
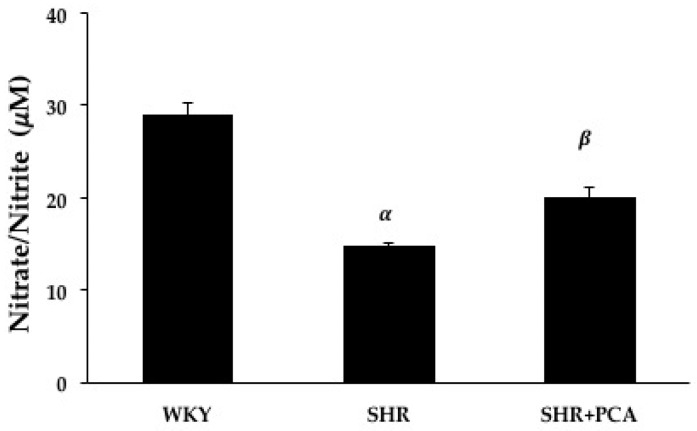
Serum nitrate/nitrite concentration among the WKY, SHR, and SHR+PCA groups. ^α^, *p* < 0.05, significant differences from WKY; ^β^, *p* < 0.05, significant differences from SHR; *n* = 8 in each group.

Figure 5 showed that serum nitrate/nitrite concentration was significantly (*p* < 0.05) reduced in the SHR group when compared with the WKY group. However, after the PCA administration, the nitrate/nitrite concentration was significantly (*p* < 0.05) increased in the SHR+PCA group when compared with the SHR group.

### 3.6. Serum MDA and Antioxidant Activities

**Figure 6:**
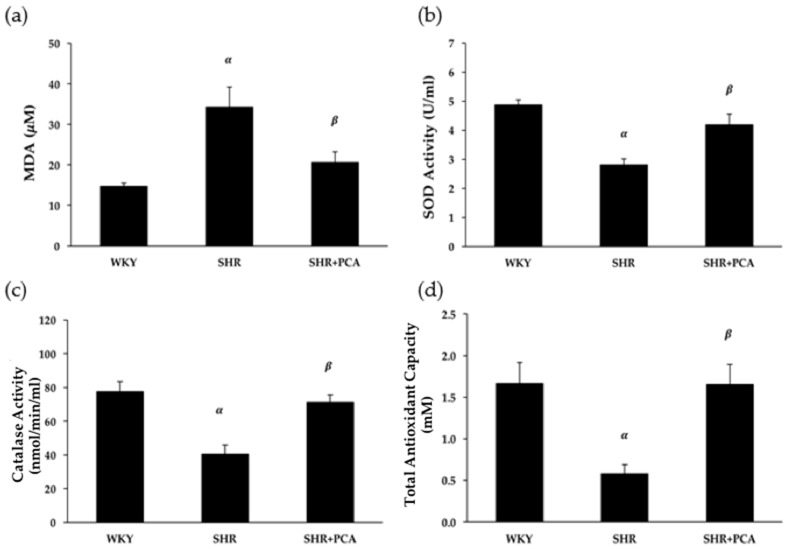
(**a**) Serum malondialdehyde (MDA) concentration, (**b**) superoxide dismutase (SOD) activity, (**c**) catalase activity, and (**d**) total antioxidant capacity among the WKY, SHR, and SHR+PCA groups. ^α^, *p* < 0.05, significant differences from WKY; ^β^, *p* < 0.05, significant differences from SHR; *n* = 8 in each group.

Serum MDA concentration and antioxidant activities, including SOD, catalase, and total antioxidants, were determined and compared among the three groups. Figure 6 showed that the SHR group had significantly (*p* < 0.05) higher MDA concentration but lower antioxidant activities, including SOD, catalase, and total antioxidants, than the WKY group. On the other hand, the PCA administration significantly (*p* < 0.05) reduced MDA concentration and enhanced these antioxidant activities in the SHR+PCA group when compared with the SHR group.

## 4. Discussion

To the best of our knowledge, the present study was the first to examine the effects of 12 weeks of PCA administration on the vasorelaxation induced by insulin and IGF-1 and antioxidant activities in aging SHR. The main findings of this study were as follows. Firstly, the PCA administration significantly improved the insulin-induced and IGF-1-induced vasorelaxation in aging SHR. However, these improvements did not reach the level of vasorelaxation in the normotensive WKY group. Secondly, in the denuded-endothelium vessels, there were no significant differences in the insulin-induced and IGF-1-induced vasorelaxation among the three groups. Thirdly, the improved vasorelaxation induced by insulin and IGF-1 in aging SHR was mainly mediated by activating the PI3K–NOS–NO signaling. Fourthly, the PCA administration significantly improved the protein levels of insulin receptors, IGF-1 receptors, p-Akt/Akt, and p-eNOS/eNOS in aging SHR. Finally, significant improvements of serum antioxidant activities, including SOD, catalase, and total antioxidants, were also found in aging SHR following the PCA administration, which partly contributed to the improved vasorelaxation.

PCA has been known to evoke multiple health benefits, such as antioxidant, anti-inflammation, anti-hyperglycemia, anti-hypertensive, and cardiovascular-protective effects [19,20,21]. Previous studies indicated that PCA extracted from the petal of *Hibiscus sabdariffa* also exhibited anti-hypertensive and cardiovascular-protective effects [19,20]. Moreover, oral administration of PCA treatments for 12 weeks significantly improved cardiac function, as shown by increased fractional shortening and left ventricular ejection fraction (LVEF) and decreased low-frequency to high-frequency ratio in streptozotocin (STZ)-induced diabetic rats [25]. Another study has shown that short-term daily supplementation with PCA (50, 100, and 200 mg/kg) dose-dependently reduced SBP and plasma H_2_O_2_ concentration, and improved antioxidant capacity in dexamethasone-induced hypertensive rats [22]. Consistent with previous studies, our investigation indicated that the 12-weeks PCA administration elicited the decreases in high blood pressure together with restoring vascular function by improving endothelium-dependent vasorelaxation induced by insulin and IGF-1 in aging SHR. Meanwhile, NO concentration was remarkably improved in aging SHR which received PCA administration. In addition to glucose metabolism, insulin and IGF-1 have vascular protective effects, including the induction of vasorelaxation, inhibition of VSMC proliferation, and anti-inflammation, mainly via stimulating the NO-dependent mechanisms in the endothelium [13]. Several reports have documented that insulin- and IGF-1-mediated vasorelaxant responses are impaired in hypertensive and diabetic animal models, which is associated with the suppressed NO bioavailability [15,27]. Consistently, the present study indicated the impairments of the insulin- and IGF-1-induced vasorelaxant responses and the decreases in NO production in aging SHR compared with the age-matched normotensive WKY. In order to clarify the NO-dependent signaling, the selective inhibitors of PI3K and NOS were administered in the insulin- and IGF-1-induced vasorelaxation. We found that, after the pre-incubation of PI3K or NOS inhibitor, these vasorelaxant differences among the three groups were absent. This suggested that the aging hypertension evoked the decreased activation of PI3K and NOS, partly resulting in the impairments of NO production and insulin- and IGF-1-induced vasorelaxation. In addition, the PCA-induced protective effects on the vasorelaxation were related to the increased activation of PI3K and NOS, resulting in the amelioration of NO production and insulin- and IGF-1-induced vasorelaxation in aging SHR.

Since the upregulation of insulin/IGF-1 receptors and downstream proteins, such as Akt and eNOS, are considered to be involved in the insulin- and IGF-1-induced vasorelaxation, we examined aortic protein expressions among the WKY, SHR, and SHR+PCA groups. We found that aging hypertension significantly decreased aortic protein expressions of insulin and IGF-1 receptors, phospho-Akt/Akt, and phospho-eNOS/eNOS, whereas the PCA administration significantly enhanced expression of these proteins in aging SHR. A previous study, in human visceral adipocytes, indicated that PCA stimulated insulin receptor substrate-1 (IRS-1) tyrosine phosphorylation and the downstream proteins, such as phosphoinositide 3-kinase binding to IRS-1 and Akt phosphorylation. Also, PCA elicited the insulin-sensitizing effects by activating adenosine monophosphate-activated protein kinase [28]. In the present study, we found that the 12-week PCA administration ameliorated the vasorelaxant responses to insulin and IGF-1 in aging SHR through the upregulation of insulin and IGF-1 receptors and downstream Akt/eNOS phosphorylation. In addition to PCA, several phytochemical compounds, such as resveratrol and curcumin, have revealed beneficial effects in reducing cardiovascular risk factors. Resveratrol lowers high blood pressure and improves endothelial function through enhancing eNOS expression and NO production. Moreover, curcumin supplementation ameliorates the impairment in endothelial-dependent dilation with aging by restoring NO bioavailability [29]. However, whether PCA induces the insulin- and IGF-1-sensitizing effects on aortic tissues needs further investigation.

It has been believed that hypertension can cause insulin resistance by altering the delivery of insulin and glucose. Conversely, insulin resistance could cause high blood pressure. In the state of insulin resistance, the insulin-stimulated NO pathway is selectively impaired and the compensatory hyperinsulinemia may activate the mitogen-activated protein kinase (MAPK) pathway, resulting in enhancement of vasoconstriction, pro-inflammation, endothelial dysfunction, increased sodium and water retention, and increases in blood pressure. Insulin resistance and hypertension commonly coexist, especially in older adults. The adverse influences of insulin resistance on blood pressure could be accentuated in an aging population [30]. Furthermore, PCA has been extensively investigated with regards to the anti-hyperglycemia activity and revealed that various doses of administration decreased blood glucose and HbA1_C_ [24,25]. Consistent with previous studies, we found that fasting blood glucose, insulin concentration, and HOMA were significantly increased in aging SHR. However, after 12 weeks of PCA intervention, these parameters were significantly reduced in aging SHR. This suggested that chronic PCA administration effectively ameliorated the aging hypertension-induced insulin resistance in aging SHR.

Oxidative stress has been considered to promote endothelial dysfunction and lead to vascular damage in hypertension. Moreover, advancing age is associated with the increased oxidative stress and reduced NO bioavailability, mediating the development of endothelial dysfunction and CVD [31,32,33]. The level of oxidative stress increases as a consequence of greater production of reactive oxygen species (ROS) without a compensatory increase in antioxidant activity. Several sources of increased ROS production include the up-regulation of the oxidant enzyme NADPH oxidase, uncoupling eNOS (due to reduced availability of the cofactor tetrahydrobiopterin), and increased mitochondrial synthesis during oxidative phosphorylation of the electron transport chain. With aging and hypertension, excessive oxidative stress could be the key mechanism mediating impaired NO bioavailability and endothelium-dependent vasorelaxation [32,33,34]. It is known that PCA administration effectively promotes antioxidant enzymatic activities and inhibits ROS generation [23]. One previous study indicated that 4-week supplementation with PCA reduced serum MDA and hydroperoxide levels, and also improved serum catalase activity, total antioxidant capacity, and glutathione concentration in deoxycorticosterone acetate (DOCA)-salt hypertensive rats [35]. Moreover, evidence has shown that PCA significantly increased the activities of glutathione peroxidase (GPx) and catalase, decreased MDA level, and normalized age--associated alterations in aged rats. This implies that PCA could induce anti-aging effects through upregulating the antioxidant system [23,36,37]. Similarly, our findings showed an increase in oxidative stress (i.e., MDA) and decreases in antioxidant activities, including SOD, catalase, and total antioxidants in aging SHR. Following the 12-week PCA administration, these impairments were significantly ameliorated in aging SHR, which partly contributed to the improved vasorelaxation. A previous study reported that PCA increased the gene expression of GPx and glutathione reductase (GR) in J774A.1 macrophages. The over-expression of glutathione-related enzymes was found by inducing c-Jun N-terminal kinase (JNK)-mediated phosphorylation of nuclear factor erythroid 2 (NF-E2)-related factor 2 (Nrf2). This suggested that PCA improved the endogenous antioxidant potential through the JNK-mediated Nrf2 activation and increased antioxidant enzyme expression [38]. It is plausible to speculate that PCA upregulates the antioxidant enzyme expression which might be responsible for modifying the enzyme activity. In this study, we first demonstrated that PCA had beneficial effects on increasing serum antioxidant activities and reducing MDA level, contributing to improved vasorelaxant responses, in aging hypertensive rats. The potential mechanisms which modify the antioxidant activity and ROS inhibition for these PCA-induced improvements in aging hypertension need to be further clarified.

Reagan-Shaw and coworkers demonstrated the dose translation from animal models to human clinical trials. The estimate of the 200 mg/kg dose in rats yields a human equivalent dose (HED) of 32.4 mg/kg for humans, which is 1944 mg in a 60-kg adult [39]. Moreover, the content of PCA varies considerably depending on the type of food, as seen in *Olea europaea* (olives), *Hibiscus sabdariffa* (roselle), *Eucommia ulmoides* (du-zhong), *Citrus microcarpa* Bunge (calamondin), and *Vitis vinifera* (white wine grapes) [21]. Further studies are recommended to clarify the effective level consumed from the daily diet.

With regard to the limitations of this study, we did not include the placebo/time-control experiments for the insulin-induced vasorelaxation, due to the limited numbers and samples of animals regulated by the IACUC. However, normotensive control (WKY) rats were included in our study, and the results demonstrated a maximal response of insulin-induced vasorelaxation of about 40%, similar to the value shown by Li et al. [40]. Moreover, McCallum and co-workers indicated that incubation with the vehicle control (without insulin) did not affect the phenylephrine-induced vasoconstriction [27]. Therefore, it is plausible to speculate that the vehicle used in final dilution of insulin may not induce significant effects on the vasoreactivity experiments, including vasorelaxation and vasoconstriction. Future work is needed to determine the placebo/time-control effects for the insulin-induced vasorelaxation. Li and co-workers also indicated that insulin produced a dose-dependent vasorelaxation with a maximal response 10–15% in aging-related hypertensive Sprague–Dawley (SD) rats [40], similar to that of the aging SHR in our study. However, the maximum dose of insulin (at 1.5 × 10^−6^ M) recorded by the Li et al. was much lower than the maximum dose used in our study. The differences of insulin-induced vasorelaxation might be caused by different rat strains and aging effects. The effects of different rat strains and age on the insulin-induced vasorelaxation need to be further investigated.

## 5. Conclusions

In conclusion, our study demonstrated that 12 weeks of PCA administration remarkably improved the endothelium-dependent vasorelaxation induced by insulin and IGF-1 in aging hypertension through enhancing the PI3K–NOS–NO pathway. Furthermore, it had strong antioxidant effects on aging hypertension, which partly contributed to the amelioration of vascular endothelial function. Based on our findings, PCA might be suggested as an alternative strategy to minimize cardiovascular disorders in the population with aging hypertension.

## Figures and Tables

**Table 1:** General characteristics.

Parameters/Groups	WKY	SHR	SHR+PCA
**Body weight (g)**	395.75 ± 8.47	393.50 ± 5.15	404.50 ± 8.85
**Heart rate (bpm)**	284.06 ± 3.90	383.19 ± 9.83 ^α^	363.38 ± 6.19 ^α,β^
**SBP (mmHg)**	121.75 ± 2.15	192.88 ± 2.28 ^α^	173.63 ± 0.88 ^α,β^
**Insulin (μg/L)**	0.34 ± 0.07	0.66 ± 0.11 ^α^	0.37 ± 0.09 ^β^
**Blood glucose (mg/dL)**	96.63 ± 2.63	121.25 ± 4.76 ^α^	110.13 ± 4.33 ^α,β^
**HOMA-IR**	2.02 ± 0.41	4.89 ± 0.82 ^α^	2.50 ± 0.54 ^β^

WKY, Wistar–Kyoto rats; SHR, spontaneously hypertensive rats; PCA, protocatechuic acid; SBP, systolic blood pressure; HOMA-IR, the homeostatic model assessment of insulin resistance. ^α^, *p* < 0.05, significant differences from WKY; ^β^, *p* < 0.05, significant differences from SHR; *n* = 8 in each group.

## Supplementary Materials

The following are available online at https://www.mdpi.com/2072-6643/11/3/699/s1, Figure S1: The SDS-PAGE and immunoblots of insulin receptor (insulin R), insulin-like growth factor-1 receptor (IGF-1 R), phospho-protein kinase B (p-Akt), protein kinase B (Akt), phospho-endothelial nitric oxide synthase (p-eNOS), endothelial nitric oxide synthase (eNOS), and actin extracted from thoracic aortas among the WKY, SHR, and SHR+PCA groups.
