# Supplementary material for: Twelve-Week Protocatechuic Acid Administration Improves Insulin-Induced and Insulin-Like Growth Factor-1-Induced Vasorelaxation and Antioxidant Activities in Aging Spontaneously Hypertensive Rats
Source: Nutrients. 2019 Mar 25;11(3):699. doi: 10.3390/nu11030699 (PMC6471824; doi:10.3390/nu11030699)
Supplement: Supplementary file 1 [file nutrients-11-00699-s001.pdf]

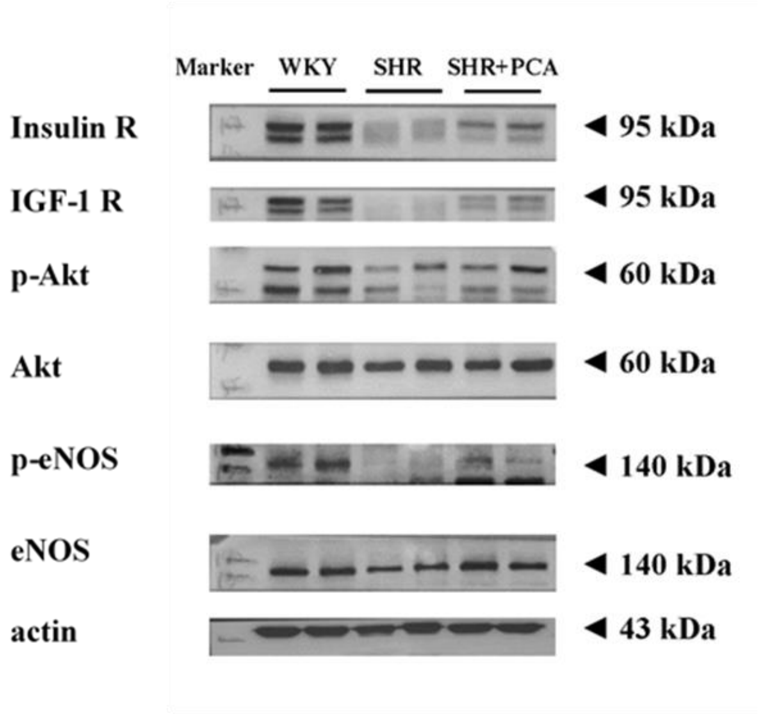

Supplementary Figure S1. The SDS-PAGE and immunoblots of insulin receptor (insulin R), insulin-like growth factor-1 receptor (IGF-1 R), phospho-protein kinase B (p-Akt), protein kinase B (Akt), phospho-endothelial nitric oxide synthase (p-eNOS), endothelial nitric oxide synthase (eNOS), and actin extracted from thoracic aortas among the WKY, SHR, and SHR+PCA groups.
